# A Novel Hybridization LC-MS/MS Methodology for Quantification of siRNA in Plasma, CSF and Tissue Samples

**DOI:** 10.3390/molecules28041618

**Published:** 2023-02-08

**Authors:** Long Yuan, Jean-François Dupuis, Kevork Mekhssian

**Affiliations:** 1 Drug Metabolism and Pharmacokinetics, Biogen, 225 Binney St., Cambridge, MA 02142, USA; 2 Altasciences, 575 Armand-Frappier Blvd., Laval, QC H7V 4B3, Canada

**Keywords:** small interfering RNA (siRNA), double-stranded oligonucleotides, hybridization, LC-MS/MS, bioanalysis, quantitative

## Abstract

Therapeutic oligonucleotides, such as antisense oligonucleotide (ASO) and small interfering RNA (siRNA), are a new class of therapeutics rapidly growing in drug discovery and development. A sensitive and reliable method to quantify oligonucleotides in biological samples is critical to study their pharmacokinetic and pharmacodynamic properties. Hybridization LC-MS/MS was recently established as a highly sensitive and specific methodology for the quantification of single-stranded oligonucleotides, e.g., ASOs, in various biological matrices. However, there is no report of this methodology for the bioanalysis of double-stranded oligonucleotides (e.g., siRNA). In this work, we investigated hybridization LC-MS/MS methodology for the quantification of double-stranded oligonucleotides in biological samples using an siRNA compound, siRNA-01, as the test compound. The commonly used DNA capture probe and a new peptide nucleic acid (PNA) probe were compared for the hybridization extraction of siRNA-01 under different conditions. The PNA probe achieved better extraction recovery than the DNA probe, especially for high concentration samples, which may be due to its stronger hybridization affinity. The optimized hybridization method using the PNA probe was successfully qualified for the quantitation of siRNA-01 in monkey plasma, cerebrospinal fluid (CSF), and tissue homogenates over the range of 2.00–1000 ng/mL. This work is the first report of the hybridization LC-MS/MS methodology for the quantification of double-stranded oligonucleotides. The developed methodology will be applied to pharmacokinetic and toxicokinetic studies of siRNA-01. This novel methodology can also be used for the quantitative bioanalysis of other double-stranded oligonucleotides.

## 1. Introduction

Therapeutic oligonucleotides are short synthetic single- or double-stranded nucleic acid strands usually in length of 15–30 base pairs. Oligonucleotides have been rapidly growing as a new class of therapeutics and are gaining increasing attention in drug discovery and development across various disease areas [1]. Antisense oligonucleotides (ASO) and small interfering RNA (siRNA) are two major types of therapeutic oligonucleotides. An ASO is a single-stranded oligonucleotide that can specifically bind to a complementary messenger RNA (mRNA) via Watson–Crick base pairings. ASO can modulate gene expression through various mechanisms, such as RNase H mediated cleavage of mRNA and steric blocking for splicing modulation. siRNA is a short double-stranded RNA that can specifically hybridize to its target RNA. siRNA usually induces the mRNA degradation via the RNA-induced silencing complex (RISC). Many disease targets lack a specific binding pocket, which is required for developing traditional small molecule drugs or protein therapeutics, and thus are considered ‘undruggable’. Oligonucleotides do not depend on a binding pocket to modulate the target, and therefore, oligonucleotide therapeutics can be developed to treat the diseases that otherwise could not be addressed [2]. There have been 13 oligonucleotide drugs approved by the US Food and Drug Administration (FDA) and/or the European Medicines Agency (EMA) by 2021 [1]. Nine out of the thirteen approved oligonucleotide drugs are ASOs and the other four are siRNA. There are also approximately 200 oligonucleotide drug candidates under clinical development, and more than 500 under preclinical development [2].

Quantitative bioanalysis of oligonucleotides plays a critical role in evaluating and understanding the pharmacokinetic (PK), toxicokinetic (TK), pharmacological, and toxicological properties of the oligonucleotide drug candidates, and the success of their drug research and development [3]. The rapid growth of oligonucleotides in drug discovery and development has resulted in significantly increased demands for quantitative bioanalysis of oligonucleotides. A bioanalytical methodology that can achieve accurate, sensitive, and reliable quantification of oligonucleotides in various biological matrices (plasma, cerebrospinal fluid, tissues, etc.) is much desired. There are three major types of approaches for oligonucleotide bioanalysis: hybridization-based immunoassays, liquid chromatography (LC) based methods, and quantitative polymerase chain reaction (qPCR) methods [4,5,6]. Conventionally, hybridization immunoassay, e.g., hybridization enzyme linked immunosorbent assay (HELISA), has been the most used methodology for the quantitative bioanalysis of oligonucleotides [4,5]. Hybridization immunoassays have the advantages of high sensitivity (single digit ng/mL or even pg/mL sensitivity), good assay performance, and minimal sample preparation. However, one major limitation of hybridization immunoassay is its lack of specificity: it is often difficult to differentiate the full-length oligonucleotide analyte from its truncated metabolites (for instance, N-1, N-2 metabolites) [4,7]. In addition, hybridization assays have relatively narrow dynamic ranges and may be affected by the presence of anti-drug antibodies in the samples [5]. qPCR is the most sensitive method for oligonucleotide bioanalysis: it can often achieve sensitivity at pg/mL level. qPCR also has the advantage of wide dynamic range. However, like hybridization immunoassays, it often lacks the specificity to mitigate the cross-reactivity with truncated metabolites. [8,9] In addition, the qPCR method requires significant method development to improve the accuracy, precision and robustness of the assay, and its application in regulated bioanalysis is limited. In recent years, liquid chromatography-tandem mass spectrometry (LC-MS/MS) has been gaining increasing attention and interests for the quantification of oligonucleotides due to its unique advantages of good specificity, wide dynamic range, and ability to simultaneously measure multiple analytes (e.g., oligonucleotide drug and its metabolites) [10,11,12,13]. However, the significantly lower sensitivity of LC-MS/MS assay compared to hybridization immunoassay limited its wider applications. To overcome the sensitivity limitation of LC-MS/MS assay, recently, a novel hybridization LC-MS/MS methodology was developed for the quantification of oligonucleotides in biological samples [14,15,16,17,18,19]. This methodology utilized a capture probe (a DNA strand complementary to the target oligonucleotide) to specifically hybridize to the oligonucleotide to achieve highly efficient sample purification. The hybridization sample preparation generated much cleaner sample extracts with improved extraction recovery compared to conventional liquid-liquid extraction (LLE) or solid phase extraction (SPE) methods. As a result, hybridization LC-MS/MS method can achieve greatly improved sensitivity that is comparable to immunoassay (below 1 ng/mL), while maintaining its advantage of high specificity. Although the truncated metabolites may still be extracted during the hybridization sample preparation process, the LC-MS/MS analysis can differentiate them from the full-length oligonucleotide analyte based on their different retention time or monitored ions. In addition, it has been demonstrated that microflow LC can further increase the sensitivity for LC-MS/MS bioanalysis of ASO [20]. The cleanness of the sample plays an important role in improving sensitivity by microflow LC: the cleaner the sample extract, the more the sensitivity improvement. Hybridization, with its highly specific and efficient sample cleanup and good recovery, is the preferred sample extraction method to be used with microflow LC to achieve the maximal sensitivity improvement.

To date, all reported hybridization LC-MS/MS methods are for the quantification of single-stranded oligonucleotides, such as small oligonucleotide [14], ASO [15,17,18,21], and microRNA [16]. No application of this methodology has been reported for the bioanalysis of double-stranded oligonucleotides (e.g., siRNA). The main challenge of applying hybridization LC-MS/MS to double-stranded oligonucleotides is the presence of a competing strand to the capture probe in the samples during the hybridization process, which could severely impact the extraction recovery. For example, if a DNA strand complementary to the antisense strand of the targe siRNA is used as the capture probe, the sense strand of the siRNA will compete with the capture probe during hybridization since they have the same sequence.

In this paper, we describe the investigation of the hybridization LC-MS/MS methodology for the quantification of double-stranded oligonucleotides in biological samples. An siRNA compound (siRNA-01) was used as the model compound. We evaluated the use of the commonly utilized DNA capture probe for the hybridization extraction. We also investigated the use of peptide nucleic acid (PNA, a synthetic oligomer that mimics DNA) as the capture probe and compared it with the DNA probe for the extraction of siRNA. For PNA, the deoxyribose phosphate backbone is substituted with a pseudo-peptide polymer consisting of N-(2-amino-ethyl) glycyl units [22]. Since PNA does not contain charged phosphate groups, and thus lacks electrostatic repulsion, the hybridization affinity between PNA and its complementary DNA strand is higher than between DNA/DNA or DNA/RNA strands [22]. The higher affinity enables the PNA capture probe to achieve higher extraction recovery of siRNA or other double-stranded oligonucleotides. Another potential advantage of a PNA probe is that the melting temperature (Tm, the temperature at which half of the oligonucleotides in solution are in the double-stranded state and half single-stranded) of a PNA/DNA or PNA/RNA duplex generally is higher than that of the corresponding DNA/DNA or DNA/RNA duplex (roughly an increase of about 1 °C per base pair). Thus, the PNA probe can anneal with the siRNA antisense strand (or sense strand) at a higher temperature to form the PNA-RNA duplex, while the competing sense strand (or antisense strand) of the siRNA remains single-stranded in the solution at the higher temperature resulting in less competition to the hybridization process. This may also help to significantly increase the hybridization extraction recovery of the PNA probe. Because of these advantages, we included the PNA probe in addition to the DNA probe in the hybridization sample preparation evaluation. We investigated and optimized the capture probe and hybridization conditions. A sensitive and reliable hybridization LC-MS/MS method was successfully developed and qualified for the quantification of siRNA-01 in various biological matrices, including plasma, cerebrospinal fluid (CSF), and tissues. To our knowledge, this is the first-time a hybridization LC-MS/MS methodology was successfully applied to quantitative bioanalysis of double-stranded oligonucleotides. The developed hybridization methodology can also be applied to other siRNAs or double-stranded oligonucleotides.

## 2. Results and Discussion

### 2.1. Selection of the Surrogate Analyte for Quantification of siRNA

It is challenging to directly analyze the intact siRNA duplex molecule as the analyte for LC-MS/MS assay because: (1) the sample preparation process will break the Watson–Crick base pairings between the siRNA strands; (2) the large size of siRNA (doubled compared to ASO) and the highly hydrophilic and negatively charged properties of siRNA make it difficult to achieve a good chromatographic behavior; and (3) the large size and highly charged properties also will result in poor sensitivity during MS/MS detection. For siRNA or other double-stranded oligonucleotides, either its antisense strand or sense strand can be analyzed as the surrogate analyte representing the intact duplex molecule [23]. The antisense strand of the siRNA is the pharmacologically active strand of the siRNA drug. Therefore, we chose to monitor AS1, the antisense strand of the siRNA-01, as the surrogate analyte for the quantification of siRNA-01. LC-MS/MS conditions were optimized using AS1 as the standard compound. The calibration curve and QC samples were prepared using siRNA-01, the intact siRNA duplex.

### 2.2. Optimization of LC-MS/MS Conditions

A Q1 full scan was conducted to obtain the MS scan ion spectra of AS1. The ions at *m/z* 629.8 (−11 charged) and 692.8 (−10 charged) were the two most abundant ions and were selected as the precursor ions. For oligonucleotides with phosphorothioate backbones, *m/z* 95 (phosphorothioate ion) is a commonly used product ion [17]. This ion also offered the best sensitivity (signal-to-noise ratio) for the analysis of AS1. After further comparison of *m/z* 629.8 and 692.8 as the precursor ions, MRM transition of *m/z* 629.8 → 95 was selected for the detection of AS1 for its better sensitivity and selectivity.

Ion-pair chromatography with a combination of alkylamine and fluoroalcohol is commonly used for the LC separation of oligonucleotides to improve the chromatographic retention and peak shape, as well as to enhance the MS response [24]. We adopted the same strategy and used LC conditions modified from previously published methods [17,21]. A combination of 10 mM DMCHA and 25 mM HFMIP (final concentration during LC separation) was used to achieve the best chromatographic separation and MS response.

### 2.3. Optimization of Hybridization Extraction Conditions

#### 2.3.1. Capture Probes

Single-stranded DNA and PNA with complementary sequences to the antisense strand of siRNA-01 were evaluated as the capture probes. To maximize the binding affinity and therefore achieving maximized extraction recovery, 100% complementary sequences were used for the DNA capture probe. Similarly, for the PNA capture probe, we used a 19-mer PNA with complementary sequence to AS1 to maximize the binding affinity. The PNA probe has two less bases than AS1: one removed each from the 3′ and 5′ end. This is to reduce the purine content and therefore improve the solubility of the probe. In addition, two lysines were added to the 3′-end of the probe to further improve its solubility. In previous work, 3′- and 5′-biotin labeled capture probes showed no difference in the performance of extraction recovery and matrix effect [17]. Therefore, in this work, we did not compare 3′- and 5′-biotin labeling and directly used 5′-biotin labeling for both the DNA and PNA capture probes.

#### 2.3.2. Temperature and Salt Concentration on the Extraction Recovery of Antisense Strand and siRNA Duplex

**Figure 1:**
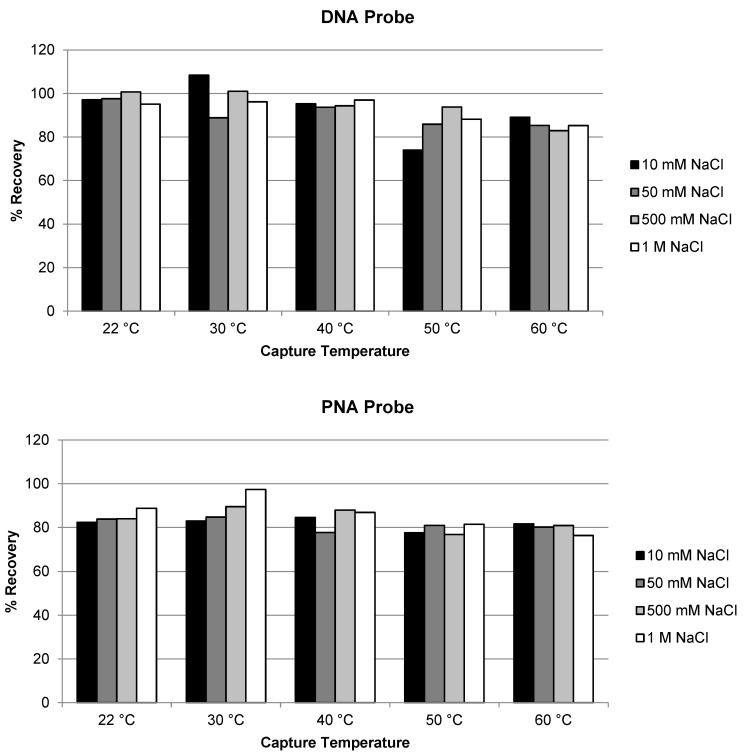
Effect of temperature and salt concentration on the recovery of AS1 using DNA probe and PNA probe.

We first evaluated the effect of temperature and salt concentration of the hybridization buffer on the extraction recovery of AS1. Hybridization temperature at room temperature (22 °C), 30, 40, 50 and 60 °C, and salt (NaCl) concentration at 10, 50, 500 and 1000 mM were compared for both the DNA and the PNA capture probe for the extraction of ULOQ samples. As shown in Figure 1, high recovery (approximately 80% or higher) of AS1 was achieved for both probes. No significant difference in recovery was observed under the different hybridization temperature and salt concentrations tested. The recovery using the DNA probe was slightly higher than using the PNA probe which may be due to experimental variation; the difference was not considered significant. The result is as expected and is consistent with previous recovery results of ASOs [17,21]: hybridization extraction of AS1, a single-stranded oligonucleotide, should be similar to the extraction of ASOs.

**Figure 2:**
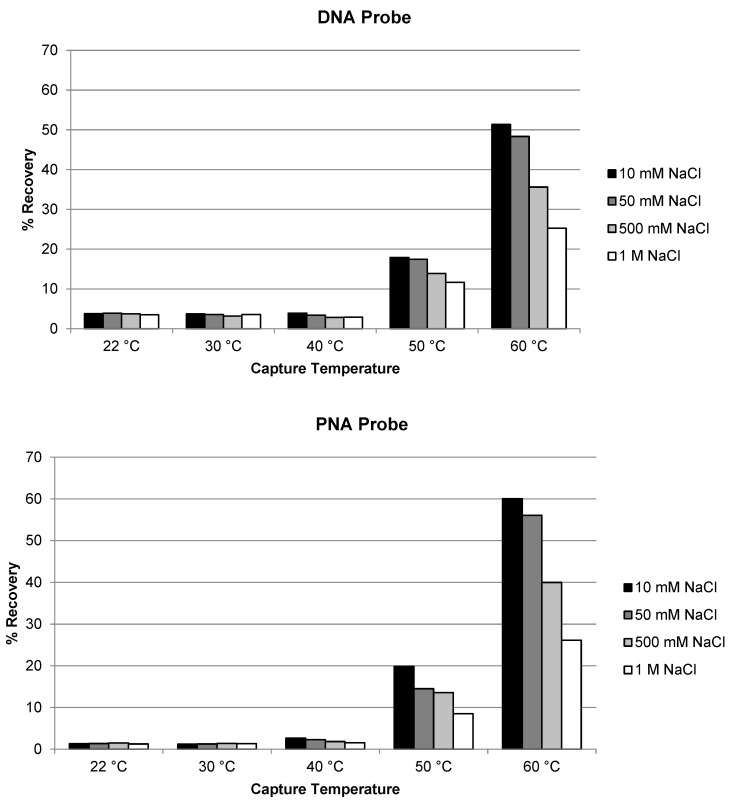
Effect of temperature and salt concentration on the recovery of siRNA-01 using DNA probe and PNA probe.

**Figure 3:**
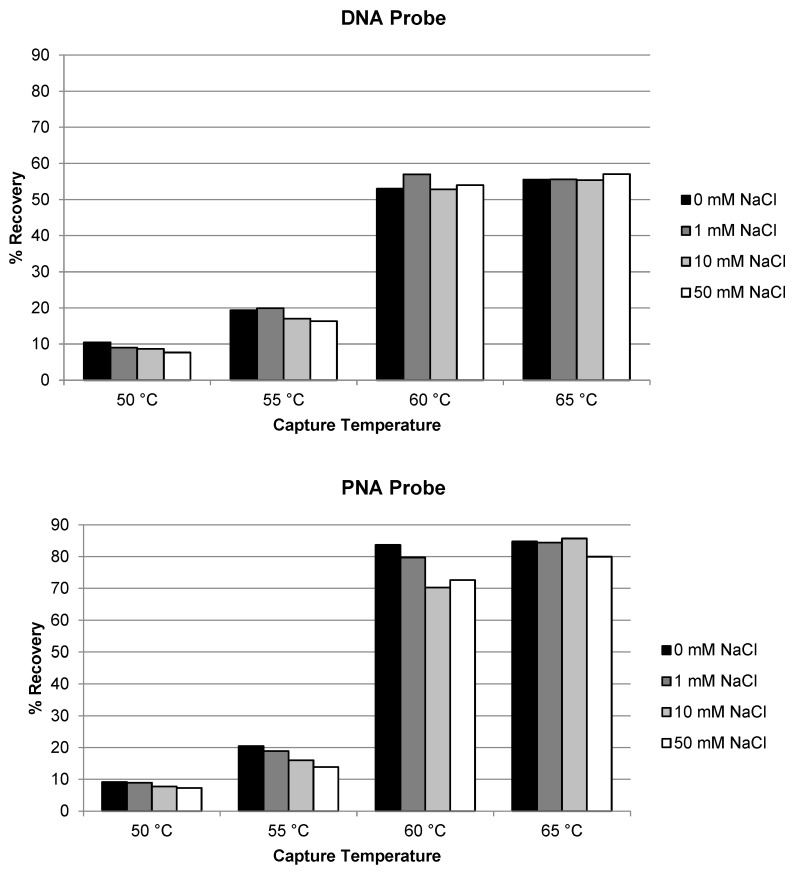
Optimization of temperature and salt concentration for the recovery of siRNA-01 using DNA probe and PNA probe.

Similar evaluation of temperature and salt concentration was also conducted for the extraction of siRNA-01 using ULOQ samples. When the capture temperature was in the range of 22 °C to 40 °C, the recoveries stayed very low at <5% for both the DNA and the PNA probes (Figure 2). The recoveries started to increase to 10–20% when the temperature was increased to 50 °C, and significantly increased to approximately 50–60% when the temperature increased to 60 °C (NaCl concentration 10 or 50 mM). At lower temperatures, siRNA-01 is predominantly present in duplex form which could not be hybridized by the capture probes. At higher temperatures, the siRNA duplex started to break, and the resulting single-stranded antisense strand annealed with the capture probes and was extracted from the samples. The higher the temperature, the more antisense strand in the single-stranded state, the better the recovery. This requirement of higher temperature for good recovery is significantly different from the extraction of single-stranded oligonucleotides (e.g., ASOs), for which room temperature usually is enough to achieve good recoveries [17,21]. The concentration of NaCl in the hybridization buffer also affected the recovery. The recovery of siRNA-01 decreased with increased salt concentration: the recovery was reduced to about half when the concentration of NaCl was increased from 10 mM to 1 M. Best recoveries were achieved at 60 °C and 10 mM NaCl for both probes. The recovery using the PNA probe was slightly (approximately 10%) higher than using the DNA probe. The temperature and salt concentration were further fine-tuned with additional temperatures at 55 and 65 °C and salt concentration at 0 and 1 mM tested. As shown in Figure 3, the condition of 65 °C and 10 mM NaCl achieved best recovery for both probes, with the recovery using PNA probe around 80% and DNA probe around 55%. Higher temperatures (75, 85, and 95 °C) were also tested. The recovery of AS1 and siRNA-01 started to decrease when the temperature was increased to 75 °C, and decreased to almost no recovery when increased to 95 °C for both probes. Therefore, the temperature of 65 °C and salt concentration of 10 mM NaCl was selected as the optimized hybridization condition for both probes for later experiments.

The higher recovery using the PNA probe than using the DNA probe is as expected, since the PNA probe has stronger binding affinity and higher melting temperature than the DNA probe. One major concern for using DNA probe is the competition between the siRNA sense strand in the samples and the DNA probe during the hybridization process, which could severely impact the extraction recovery. It was surprising that the DNA probe also achieved reasonable recovery (around 50%) of the siRNA duplex at higher temperature (65 °C). There are two main possible reasons for this. First, the amount of the DNA capture probe is approximately 20-fold excess compared to the ULOQ of the siRNA, and even more excess for lower concentration samples. The much excessive amount of capture probe ensured that the antisense strand in the samples mainly hybridized to the capture probe, and only a small percentage may hybridize to the competing sense strand. Second, the capture probe is immobilized on the surface of the streptavidin beads. This solid support (beads-based) approach may offer better hybridization efficiency for the capture probe with the antisense strand than the competing sense strand in the solution.

#### 2.3.3. DNA Probe Concentration and Incubation Time on the Recovery

**Figure 4:**
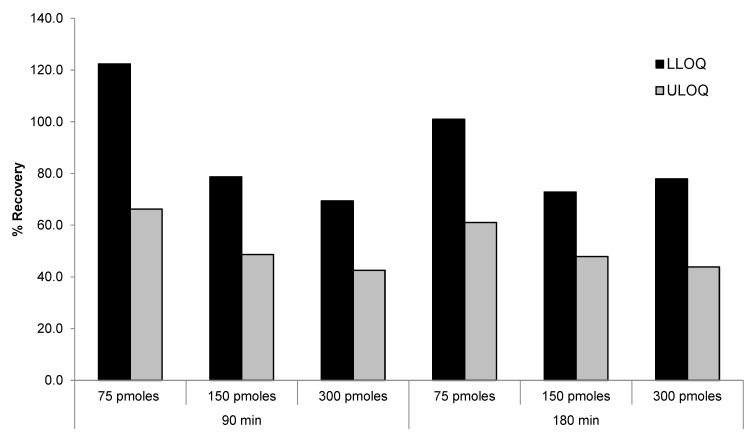
Effect of DNA probe concentration and incubation time on the recovery of siRNA-01.

The linearity of the method was tested with a calibration curve of siRNA-01 in monkey plasma (2.00–1000 ng/mL) extracted using DNA probes at 10, 37.5, and 75 pmoles/sample. Slight lack of linearity was observed at the high end of the curve indicating that the recoveries of high concentration standard samples were lower compared to that of the low concentration standards. To improve the recovery of DNA probe for high concentration samples, we tested the recoveries of LLOQ and ULOQ samples using higher probe concentrations (150 and 300 pmoles/sample vs. 75 pmoles/sample), as well as longer incubation time (180 min vs. 90 min). As shown in Figure 4, the recovery of ULOQ samples were approximately 30% lower than the recovery of LLOQ samples using probe concentration of 75 pmoles/sample and 90 min incubation time. Increasing the probe concentration to 150 and 300 pmoles/sample did not improve the recovery of ULOQ samples. On the contrary, there was a trend of decreased recovery with the increase of probe concentration. When the incubation time was increased from 90 min to 180 min, there was no improvement in recoveries either under all the tested conditions. This may because the probe concentration of 75 pmoles/sample is already approximately 20-fold in excess to the ULOQ sample and therefore high enough for maximizing the hybridization recovery. Further increase of the probe concentration may cause increased loss of the analyte due to non-specific binding to the probes/beads.

#### 2.3.4. PNA Probe vs. DNA Probe

**Figure 5:**
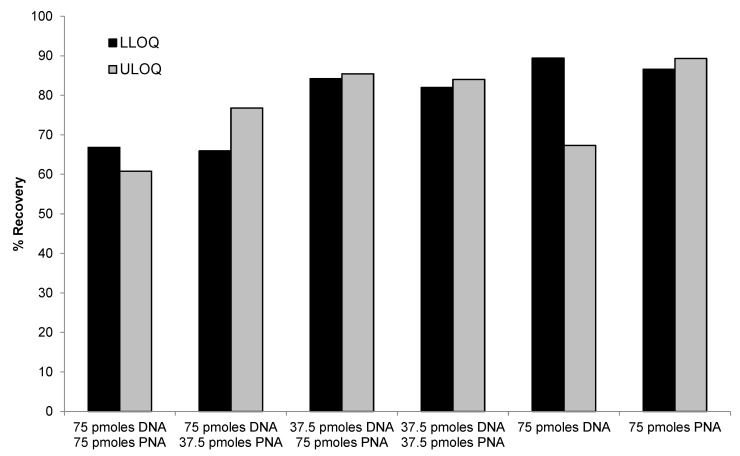
Comparison of different combination of DNA and PNA probes for recovery of siRNA-01.

PNA probe alone (75 pmoles/sample), DNA probe alone (75 pmoles/sample), or mixtures of various concentrations of DNA (37.5 or 75 pmoles/sample) and PNA probes (37.5 or 75 pmoles/sample) were compared for the recovery of LLOQ and ULOQ samples. As shown in Figure 5, similar recovery between ULOQ and LLOQ samples were achieved using PNA alone or mixtures of PNA and DNA probes. Lower recovery of ULOQ samples was observed when using DNA probe alone, which was consistent with previous results. Best performance (>85% recovery for both LLOQ and ULOQ) was achieved using 75 pmoles/sample of PNA probe. The use of a mixture of 37.5 or 75 pmoles/sample of PNA probe with 37.5 pmoles/sample of DNA probe also achieved satisfactory recovery (>80%). These results indicated that the PNA probe was more effective than the DNA probe in extracting high concentration samples, which is consistent with our hypothesis that the stronger hybridization affinity and higher melting temperature of the PNA probe may help to improve the recovery.

To confirm the performance of the PNA probe, a calibration curve (2.00–1000 ng/mL) and QCs at 2.00, 6.00, 500, and 750 ng/mL in monkey plasma were extracted using 75 pmoles/sample PNA probe or 37.5 pmoles/sample each of PNA and DNA probes. The assay showed good linearity with similar accuracy and precision under both extraction conditions (Table 1). The use of 75 pmoles/sample of PNA probe was selected as the final method for its simplicity.

### 2.4. Equivalence of the Assay for the Analysis of siRNA-01 Duplex and Single-Stranded AS1

In in vivo samples, the siRNA may exist in both duplex and single-stranded form. It is critical that the developed method can accurately quantify both forms in the samples. Theoretically, during sample preparation, the duplex form of the siRNA in the samples will be converted to the single-stranded form after incubation at high temperature which will then be hybridized by the capture probe and then further processed for LC-MS/MS analysis. As a result, the hybridization LC-MS/MS method should be able to accurately quantify both the duplex and single-stranded form equivalently. To investigate if this holds true, we prepared low and high QCs of siRNA-01, AS1, and 1:1 mixture of siRNA-01 and AS1 in monkey plasma (concentrations of AS1 were normalized to equal molar of the siRNA-01 concentrations). A siRNA-01 calibration curve (2.00–1000 ng/mL of siRNA-01 in plasma) was used to analyze all the prepared QC samples. As shown in Table 2, the accuracy and %CV of siRNA-01, AS1 and siRNA-01/AS1 mixture QCs at low and high concentrations were all within ±15%, demonstrating that the developed hybridization LC-MS/MS method can accurately quantify both the duplex and single-stranded form of siRNA across the assay range.

### 2.5. Qualification and Performance of the Hybridization LC-MS/MS Method

To support a toxicity study of siRNA-01 in monkeys, the optimized method was qualified for the quantification of siRNA-01 in monkey plasma, CSF, and tissue homogenates prepared from various types of tissues. Since similar method performances were achieved for monkey plasma, CSF, and tissue homogenate samples, the results of the monkey plasma method were described and discussed below as representative.

#### 2.5.1. Accuracy, Precision, and Curve Linearity

Accuracy and precision of the optimized method for siRNA-01 in monkey plasma were assessed for QCs at four concentration levels in two runs. Table 3 summarizes the accuracy and precision data of siRNA-01 QCs in monkey plasma. Based on the three levels of analytical QCs (low, mid, and high), the within-run precision was within 9.3% CV, and the accuracy was 92.1–99.3% of the nominal concentration for siRNA-01, all well within the 15% acceptance criteria. A quadratic 1/x^2^ weighted regression model (y = ax^2^ + bx + c) provided the best statistical fit for siRNA-01 over the range of 2.00 to 1000 ng/mL with coefficient of determination (R^2^) ≥ 0.9932 in both runs. All the calibration standards in the runs passed the acceptance criteria of within ± 15% of nominal concentrations (within ± 20% nominal at the LLOQ level). These results demonstrated the good accuracy and precision of the method for the analysis of siRNA-01 in monkey plasma.

#### 2.5.2. Specificity and Sensitivity

**Figure 6:**
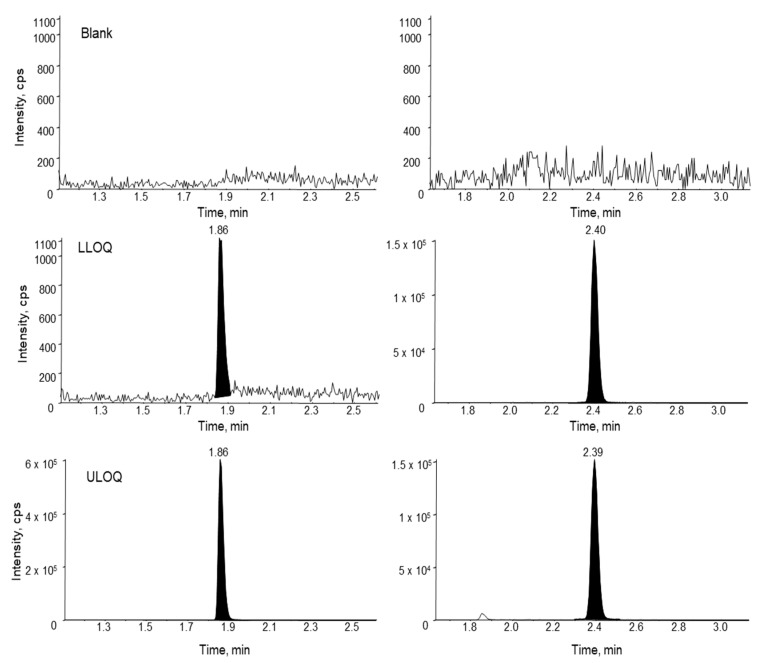
Representative MRM chromatograms of siRNA-01 (**left**) and the internal standard ASO-002 (**right**) in a blank monkey plasma, a monkey plasma spiked with the analyte at the LLOQ concentration (2.00 ng/mL), and ULOQ (1000 ng/mL) in monkey plasma.

Assay specificity was evaluated using three different lots of monkey plasma. No significant interfering peaks were observed at the retention time of either the analyte or the IS for all the different lots of blank monkey plasma samples, indicating good specificity of the assay. Representative MRM chromatograms of siRNA-01 in a blank monkey plasma, a LLOQ, and a ULOQ samples are presented in Figure 6. The signal-to-noise ratio of the LLOQ sample was much greater than 5. The intra-run accuracy and precision all met the 20% acceptance criteria (Table 3), which demonstrated the establishment of LLOQ at 2.00 ng/mL in monkey plasma. The same LLOQ of 2.00 ng/mL was also established for siRNA-01 in treated monkey CSF and tissue homogenates.

#### 2.5.3. Extraction Recovery and Matrix Effect

The extraction recovery of siRNA-01 was determined at 6.00 ng/mL (low QC) and 750 ng/mL (high QC) by comparing the analyte to IS response ratios in monkey plasma samples, which were spiked with the analyte before the extraction, with those spiked after the extraction. The recovery of siRNA-01 at low and high QC was 93.5%, and 86.3%, respectively, indicating good extraction recovery of the analyte from plasma across the assay range.

The matrix effect, which is the ion suppression or enhancement of the analyte by coeluting matrix components, is expressed as matrix factor (MF). The MF was determined by calculating the ratio of the analyte response in plasma extract spiked post-extraction to the response of analyte spiked in elution solution. The MF of the IS was calculated similarly. The IS-normalized MF was determined by dividing the analyte MF by the IS MF. Three different lots of monkey plasma were evaluated in triplicate for the MF at 6.00 and 750 ng/mL. As shown in Table 4, the MF of the analyte at 6.00 ng/mL was 0.92–1.02, the MF of the IS was 0.96–1.03, and the IS-normalized MF was 0.92–1.03. The %CV for the IS-normalized MF of three lots of plasma was 4.1%. At 750 ng/mL, the MF of the analyte, the IS, and the IS-normalized MF were 0.95–1.08, 0.92–1.02, and 0.98–1.08, respectively, and the %CV for the IS-normalized MF was 3.9%. These results demonstrated that there was minimal matrix effect on the analysis of the analyte indicating a highly selective extraction of the analyte by this method resulting in very clean sample extract.

#### 2.5.4. Stability

The bench-top (room temperature), frozen storage (−80 °C), and freeze–thaw stability of siRNA-01 in monkey plasma were evaluated. siRNA-01 was stable in monkey plasma for at least 8 h at room temperature, 7 days at −80 ºC, and after 3 freeze–thaw cycles.

#### 2.5.5. Metabolite Interference

siRNA duplex compounds usually are metabolized by endonucleases and exonucleases ubiquitously presented in vivo and form truncated metabolites of its antisense or sense strand [23]. To ensure there is no interference from the potential metabolites (e.g., n-1 truncated metabolite) to the quantification of siRNA-01, the putative 3′ n-1 metabolite was synthesized and assessed in a metabolite interference test. As the expected metabolite level was <20% of the intact antisense strand and the antisense strand would be the most abundant species present in the samples, the 3′ n-1 metabolite was spiked into siRNA-01 low QC (6.00 ng/mL) and high QC (750 ng/mL) samples at 20% of the corresponding siRNA-01 concentrations. The spiked QCs were analyzed in quadruplicate, and the average %Bias was at −5.9% and −1.6%, and the %CV at 6.0% and 3.1% for low and high QC, respectively, which indicated the absence of metabolite interference. We did not test n-2, n-3, or other shorter truncated metabolites in this work. These metabolites usually will not cause interference to the analysis of the intact antisense strand [21], since their much smaller size would make them easily differentiated from the analyte by LC-MS/MS.

#### 2.5.6. Dilution Integrity and Nonspecific Binding Test

Dilution integrity of the assay was evaluated by analyzing dilution QC samples (2000 ng/mL in monkey plasma, *n* = 4) extracted with 10-fold dilution. The accuracy was 107.1% of the nominal concentration, and the precision (%CV) was 5.8%, all within 15% acceptance criteria, demonstrating satisfactory dilution integrity.

Nonspecific binding loss of siRNA-01 in monkey plasma was tested by analyzing the low QC samples (6.00 ng/mL, *n* = 4) after 5 × transfers in polypropylene tubes. There was no decrease (%difference 2.9%) in siRNA-01 concentration (analyte/IS response ratio) for the QCs after 5 × transfers compared to the QCs without any transfer, indicating no loss of siRNA-01 due to nonspecific binding.

### 2.6. CSF Assay

Non-specific binding (NSB) loss of analyte is a well-known challenge for the analysis of oligonucleotides, especially for a protein deficient matrix such as CSF. To prevent the potential NSB loss during sample preparation and analysis, we used a previously developed anti-adsorption treatment solution [21], which is a combination of nonionic surfactant and protein (2% Tween 80 and 100 mg/mL BSA in TBS), to treat the CSF samples 1:1 (*v/v*). Due to the limited availability of monkey CSF, we used artificial CSF (aCSF) as a surrogate matrix for the CSF assay. The aCSF was treated the same way using the anti-adsorption treatment solution to prevent the NSB and minimize the matrix difference between aCSF and real CSF samples. Nonspecific binding loss of siRNA-01 in treated monkey CSF and aCSF was tested by analyzing the low QC samples after 5 × transfers in polypropylene tubes. No nonspecific binding loss of siRNA-01 was observed after 5 × transfers (%difference −1.9% and 0.7% in treated monkey CSF and treated aCSF, respectively).

### 2.7. Tissue Assay

**Figure 7:**
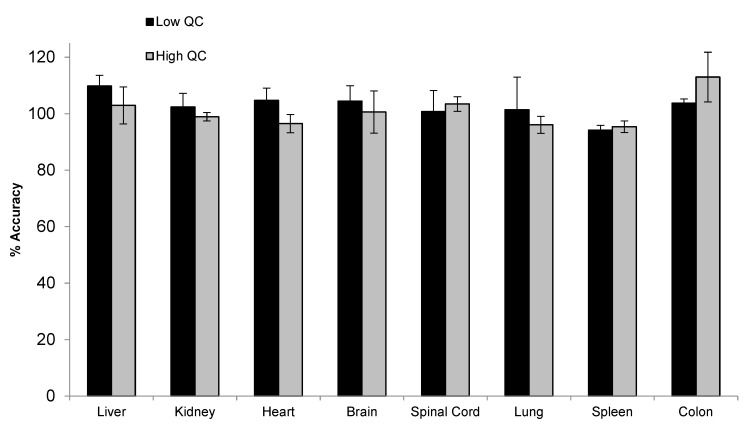
Accuracy and precision of siRNA-01 in monkey liver, kidney, heart, brain, spinal cord, lung, spleen, and colon homogenates (calibration curve prepared in a combined tissue homogenate).

To support the toxicity study, tissue assays need to be developed and qualified for the quantification of siRNA-01 in eight different types of tissues, including brain, kidney, liver, heart, lung, spleen, colon, and spinal cord. For traditional LBA or LC-MS/MS approaches, due to the significantly different matrix effect between different tissues, multiple methods may need to be developed for individual tissues and samples may be analyzed separately. Hybridization LC-MS/MS, with its highly selective and efficient sample extraction, usually generates clean extract with minimal matrix effect. Thus, a surrogate matrix approach can be used for the bioanalysis of different types of tissues [21]. Here, we used a combined tissue homogenate from different tissues (equal parts brain, kidney, liver, heart, lung, spleen, and colon tissue homogenates) as the surrogate matrix to prepare the calibration curve. The method was qualified with QCs prepared in individual type of tissues. Initially, monkey tissues were homogenized in a ratio of 1:12.5 (*w/v*) using the homogenization buffer (20 mM Tris, 20 mM EDTA, 100 mM NaCl, 0.5% NP-40, pH 8.0). However, variabilities were still observed between different types of tissue, indicating the presence of matrix effect. After further diluting the tissues during the homogenization step using a 1:37.5 ratio, good accuracy and precision were achieved for siRNA-01 in all different types of tissue homogenates over the range of 2.00–1000 ng/mL. Matrix effect (matrix factor) was assessed using low (6.00 ng/mL) and high (750 ng/mL) QC samples prepared in individual tissue homogenate (three difference lots, each lot in triplicates). The mean IS normalized MF was 0.97 (%CV 3.9%) and 1.04 (%CV 5.4%) for low and high QC, respectively. Figure 7 showed the accuracy and precision results of individual tissue QCs at low and high concentrations. The mean accuracy (%Bias) was within ±13.0% and %CV (*n* = 3) was within 11.4% for all eight different types of tissue homogenate QCs at both low and high concentrations. The results demonstrated that the tissue assay had minimal matrix effect and excellent accuracy and precision. This surrogate matrix method was successfully qualified for the analysis of eight different types of tissues which greatly saved the resources and improved the efficiency.

## 3. Materials and Methods

### 3.1. Chemicals, Reagents, Materials, and Instrumentation

The analyte siRNA-01, the antisense strand of siRNA-01 (AS1, a 21-mer oligonucleotide), the analogue internal standard (IS) ASO-002, and 3′ n-1 truncated metabolite of AS1 were proprietary compounds obtained from Biogen (Cambridge, MA, USA) and its collaborator (see Table 5 for the basic compound information). The biotinylated DNA capture probe (5′-BiotinTEG-DNA, full sequence reverse-complementary to AS1) was synthesized by Integrated DNA Technologies (Coralville, IA, USA). The biotinylated peptide nucleic acid (PNA) capture probe (5′-Biotin-OO-PNA-KK, 19-mer reverse-complementary to AS1) was synthesized by PNA Bio (Newbury Park, CA, USA).

Acetonitrile (ACN), methanol (MeOH), N,N-dimethylcyclohexylamine (DMCHA), 1,1,1,3,3,3-hexafluoro-2-methyl-2-propanol (HFMIP), ethylenediaminetetraacetic acid (EDTA), 10 N sodium hydroxide (NaOH), tris(Hydroxymethyl)aminomethane (Tris), Tween 20, sodium chloride (NaCl), and DL-dithiothreitol (DTT) were obtained from MilliporeSigma (Burlington, MA, USA). Clarity OTX Lysis-Loading Buffer v 2.0 was purchased from Phenomenex (Torrance, CA, USA). Proteinase K, Dynabeads MyOne Streptavidin C1, and Blocker BSA in TBS (10×) Concentrate (100 mg/mL) were obtained from Thermo Fisher Scientific (Waltham, MA, USA). NONIDET P40 (NP-40) was purchased from Accurate Chemical & Scientific (Carle Place, NY, USA). LoBind Eppendorf 1 mL round bottom 96-well plates were obtained from Eppendorf (Enfield, CT, USA). Control rat plasma (K_2_EDTA), monkey plasma (K_2_EDTA), monkey CSF, and monkey tissues (liver, brain, kidney, heart, spleen, colon, lung, and spinal cord) were purchased from BioIVT (Westbury, NY, USA). Artificial CSF (aCSF) was purchased from Tocris Bioscience (Toronto, ON, Canada).

A Shimadzu Nexera X2 UHPLC system (Shimadzu, Columbia, MD, USA) equipped with three LC-30AD pumps was used for the LC separation. A Sciex TripleQuad 6500+ mass spectrometer (Sciex, Framingham, MA, USA) with Analyst software v 1.6.3 was used for the mass spectrometric detection. A KingFisher Flex Purification System (ThermoFisher Scientific, Waltham, MA, USA) was used for the hybridization sample preparation.

### 3.2. Preparation of Calibration Standard and Quality Control Samples

The intact siRNA-01 (duplex of the full-length oligonucleotides) was used for the preparation of calibration standard (STD) and quality control (QC) samples. The siRNA-01 stock solution (2958 µM in PBS) was diluted in ASO diluent (25 mM HFMIP, 10 mM DMCHA, 100 μM EDTA, and 0.05% rat plasma K2EDTA in water:ACN 90:10% *v/v*) to prepare the intermediate solution (1000 μg/mL). The intermediate solution was appropriately diluted in ASO diluent to prepare ten STD spiking solutions (200–100,000 ng/mL) and four QC spiking solutions (200, 600, 50,000, and 75,000 ng/mL). The ASO-002 IS stock solution was prepared at 500 μg/mL in water, which was further diluted in ASO diluent to obtain 20.0 ng/mL IS working solution (ISWS).

To prepare STD samples in monkey plasma, the STD spiking solutions were diluted 100-fold with blank monkey plasma to prepare STDs from 2.00 ng/mL (lower limit of quantitation, LLOQ) to 1000 ng/mL (upper limit of quantification, ULOQ). Similarly, QC samples, including LLOQ QC at 2.00 ng/mL, low QC at 6.00 ng/mL, mid QC at 500 ng/mL, and high QC at 750 ng/mL, were prepared in monkey plasma. A dilution QC at 2000 ng/mL was prepared by appropriate dilution of the intermediate solution.

Monkey tissue homogenate was prepared by homogenizing the individual tissues (brain, kidney, liver, heart, lung, spleen, colon, and spinal cord) in homogenization buffer (20 mM Tris, 20 mM EDTA, 100 mM NaCl, 0.5% NP-40, pH 8.0) in a ratio of 1:37.5 (*w/v*). A combined tissue homogenate was prepared by mixing brain, kidney, liver, heart, lung, spleen, and colon tissue homogenates at 1:1:1:1:1:1:1 ratio. The STD/QC samples in monkey CSF, artificial CSF (aCSF), and combined or individual tissue homogenate were prepared similarly to the preparation of plasma STD/QC samples using the corresponding matrices. For the preparation of monkey CSF and aCSF samples, after the spiking of the analyte, the samples were treated with equal volume (1:1, *v/v*) of the anti-adsorptive agent (2% Tween 80 in 100 mg/mL BSA in TBS).

### 3.3. Sample Preparation

Streptavidin magnetic beads preparation: Dynabeads MyOne Streptavidin C1 magnetic beads (15 µL per sample) were washed 3 times with the washing buffer (5 mM Tris, 1 M NaCl, 0.5 mM EDTA and 0.05% Tween 20 in water). The beads were resuspended to the same buffer and incubated with capture probe (approximately 0.5 nmol probe per 100 µL of beads) at room temperature for 1 h. After incubation, the coated beads were washed 3 times and resuspended to the original volume of washing buffer.

Hybridization sample extraction: add samples (50 µL of plasma or tissue homogenate or 100 µL of treated CSF or aCSF), 100 µL of Lysis Buffer (Clarity OTX Lysis-Loading Buffer), 180 µL of Digestion Buffer (100 mM Tris, 250 mM NaCl, 10 mM EDTA and 10 mM DTT in water, prepared fresh daily), and 20 µL of Proteinase K (20 mg/mL) into wells of a 96-well plate. Briefly centrifuge the plate and then incubate for 120 min at 65 °C using a plate shaker at a speed of 850 rpm. Then, add 400 µL of Capture Buffer (10 mM Tris, 10 mM NaCl, 1 mM EDTA and 0.05% Tween 20 in water) and 15 µL of prepared streptavidin beads to the samples. Incubate the sample plate for 90 min at 65 °C. Then, on a KingFisher Flex Purification System, wash the samples twice at room temperature using 500 µL of 10 mM Tris, 10 mM NaCl, 1 mM EDTA and 0.05% Tween 20 in water. Then, add 125 µL of ISWS to the plate and incubate at 95 °C for 20 min for the elution. Transfer the sample eluent to a LoBind Eppendorf plate, centrifuge at 4612× *g* for 5 min, and then submit for LC-MS/MS analysis.

### 3.4. UHPLC-MS/MS Conditions

Chromatographic separation was achieved using an Acquity UPLC Oligonucleotide BEH C18 column (2.1 × 50 mm, 1.7 µm particle size; Waters, Milford, MA, USA) coupled with an Acquity BEH C18 guard column (VanGuard 2.1 × 5 mm, 1.7 μm particle size; Waters, Milford, MA, USA). The column temperature was set at 70 °C. The mobile phases consisted of water (mobile phase A), ACN/water 90/10 (mobile phase B) and 125 mM HFMIP, 50 mM DMCHA in ACN/water 50/50 (mobile phase C). The gradient and flow-rate conditions are presented in Table 6. The total run time was 7 min. The injection volume was 10 µL.

The mass spectrometric detection was conducted in electrospray negative ionization mode using the following parameters: ion spray voltage −4500 V; temperature 550 °C; curtain gas 30 units; ion source gas 1, 80 units; ion source gas 2, 60 units; dwell time 250 msec for AS1 and 50 msec for the IS; declustering potential −75 V for the analyte and −65 V for the IS; collision energy −140 eV for AS and −150 eV for the IS, respectively. The monitored multiple reaction monitoring (MRM) transitions were *m/z* 629.8 → 95 for AS1 and *m/z* 879.5 → 95 for ASO-002, the IS.

## 4. Conclusions and Future Perspectives

A hybridization LC-MS/MS method was successfully developed and qualified for the quantification of siRNA-01, an investigational siRNA drug candidate, in monkey plasma, CSF, and tissue homogenate over the range of 2.00–1000 ng/mL. The use of the PNA probe as the capture probe achieved satisfactory recovery (around 90%) for the hybridization extraction of siRNA-01. This is the first report of applying hybridization methodology for the quantitative bioanalysis of a double-stranded oligonucleotide. The developed methodology will be used to support pharmacokinetic, toxicokinetic, and biodistribution studies of siRNA-01 in monkeys. The methodology can also be applied to the bioanalysis of other siRNAs or double-stranded oligonucleotides.

With the rapid growth in oligonucleotide therapeutics, sensitive and reliable bioanalytical methods are in increasing demand to characterize the PK, TK, and biodistribution of oligonucleotide drug candidates and therefore support their research and development. Hybridization LC-MS/MS combines the advantages of hybridization immunoassay and LC-MS/MS, and has been proven to be able to provide accurate, sensitive and specific bioanalysis of single-stranded oligonucleotides. In this work, the application of this strategy to double-stranded oligonucleotides, such as siRNA, has also been successfully demonstrated. Thus, hybridization LC-MS/MS as a novel platform is able to be more widely applied to the bioanalysis of all types of oligonucleotides, including ASO, siRNA, or other oligonucleotides, in various biological matrices. In combination with other advanced technologies in chromatography and mass spectrometry, the sensitivity and selectivity/specificity of hybridization LC-MS/MS methods can be further improved (e.g., the use of microflow LC to increase the sensitivity and high-resolution mass spectrometry to improve specificity). In addition, since oligonucleotides are composed with the same nucleic acid bases or modifications, they have similar properties. The developed method and the learnings for one oligonucleotide can be easily transferred/applied to other oligonucleotides. This will significantly save the time and efforts and increase the efficiency of method development. This unique advantage, along with its good sensitivity and selectivity, makes hybridization LC-MS a superior platform for wider application in oligonucleotide bioanalysis, covering both single-stranded and double-stranded oligonucleotides.

## Figures and Tables

**Table 1:** Comparison of different capture probes for the analysis of siRNA-01 QCs in monkey plasma.

PNA Probe 75 pmols/Sample	LLOQ QC 2.00 ng/mL	Low QC 6.00 ng/mL	Mid QC 500 ng/mL	High QC 750 ng/mL
Measured Conc.	2.14	5.78	502.18	758.45
	2.03	6.32	417.41	726.41
	1.79	6.21	446.34	726.46
	1.95	6.00	472.78	788.82
Mean	1.98	6.08	459.68	750.03
S.D.	0.15	0.24	36.25	29.94
N	4	4	4	4
% CV	7.5	4.0	7.9	4.0
% Nominal	98.7	101.3	91.9	100.0
PNA and DNA Probes 37.5 pmols Each/Sample	LLOQ QC 2.00 ng/mL	Low QC 6.00 ng/mL	Mid QC 500 ng/mL	High QC 750 ng/mL
Measured Conc.	1.98	6.25	526.62	751.12
	2.09	6.07	457.77	745.37
	2.24	6.04	533.94	752.48
	2.01	6.42	456.61	742.90
Mean	2.08	6.19	493.73	747.97
S.D.	0.12	0.18	42.31	4.57
N	4	4	4	4
% CV	5.6	2.8	8.6	0.6
% Nominal	103.9	103.2	98.7	99.7

**Table 2:** Equivalence of the hybridization LC-MS/MS assay for the quantification of siRNA-01 and AS1.

	siRNA-01 Low QC 6.00 ng/mL	AS1 Low QC 6.00 ng/mL	siRNA-01 + AS1 Low QC 3.00 + 3.00 ng/mL	siRNA-01 High QC 750 ng/mL	AS1 High QC 750 ng/mL	siRNA-01 + AS1 High QC 375 + 375 ng/mL
	6.46	6.27	5.80	696.41	752.41	733.57
	6.55	5.99	5.99	677.39	813.93	705.49
	6.92	6.50	5.10	782.65	761.66	680.36
	5.62	5.53	5.41	799.26	759.01	822.64
Mean	6.39	6.07	5.58	738.93	771.75	735.52
S.D.	0.55	0.42	0.40	60.95	28.39	62.02
*n*	4	4	4	4	4	4
% C.V.	8.6	6.9	7.2	8.2	3.7	8.4
% Nominal	106.5	101.2	92.9	98.5	102.9	98.1

**Table 3:** Accuracy and precision of siRNA-01 QCs in monkey plasma.

Run 1	LLOQ QC 2.00 ng/mL	Low QC 6.00 ng/mL	Mid QC 500 ng/mL	High QC 750 ng/mL
Measured Conc.	1.98	5.87	527.11	681.34
	1.94	6.50	441.75	674.75
	2.05	5.91	464.04	756.63
	2.29	5.56	469.18	753.45
Mean	2.06	5.96	475.52	716.54
S.D.	0.16	0.40	36.40	44.55
*n*	4	4	4	4
% CV	7.7	6.6	7.7	6.2
% Nominal	103.1	99.3	95.1	95.5
Run 2	LLOQ QC 2.00 ng/mL	Low QC 6.00 ng/mL	Mid QC 500 ng/mL	High QC 750 ng/mL
Measured Conc.	1.78	5.83	493.31	746.88
	1.82	5.06	460.57	754.49
	1.72	5.12	471.69	721.32
	1.75	6.09	492.21	702.19
Mean	1.77	5.53	479.45	731.22
S.D.	0.04	0.51	16.04	24.00
*n*	4	4	4	4
% CV	2.4	9.3	3.3	3.3
% Nominal	88.3	92.1	95.9	97.5

**Table 4:** Matrix effect of the analysis of siRNA-01 in monkey plasma.

	Low QC (6.00 ng/mL)			High QC (750 ng/mL)		
	Analyte Matrix Factor	IS Matrix Factor	IS-Normalized Matrix Factor	Analyte Matrix Factor	IS Matrix Factor	IS-Normalized Matrix Factor
Lot 1	0.92	0.97	0.95	1.00	1.01	0.99
	0.95	1.02	0.93	0.98	0.98	1.00
	1.01	1.01	1.00	0.94	0.95	0.99
Lot 2	0.92	1.00	0.92	1.04	1.02	1.02
	0.97	1.00	0.97	0.95	0.92	1.03
	0.99	0.96	1.03	1.08	1.00	1.08
Lot 3	1.02	1.01	1.01	0.99	0.98	1.01
	0.98	1.03	0.95	0.98	1.00	0.98
	0.94	1.01	0.93	1.04	0.96	1.08
Mean			0.97			1.02
S.D.			0.040			0.040
*n*			9			9
% C.V.			4.1			3.9

**Table 5:** siRNA-01 antisense strand, AS1, and its internal standard, ASO-002.

Name	MW (kDa)	Sequence Length	Chemistry
AS1	6.9	21	Mixed backbone with 2′-OMe and 2′-F modification
ASO-002	7.9	20	Uniform MOE with PS backbone

2′-OMe: 2′-O-methylation. MOE: 2′-O-(2-Methoxyethyl)-oligoribonucleotides. PS: phosphorothioate linkage.

**Table 6:** LC gradient and flow rate conditions.

Time (min)	Module	Events	Parameters
0	Pumps	Pump B Conc.	0
0	Pumps	A/B Total Flow	0.400
0	Pumps	Pump C Flow	0.100
0.3	Pumps	Pump B Conc.	0
3.0	Pumps	Pump B Conc.	25
3.0	Pumps	A/B Total Flow	0.400
3.0	Pumps	Pump C Flow	0.100
3.1	Pumps	Pump C Flow	0
3.2	Pumps	Pump B Conc.	100
3.2	Pumps	A/B Total Flow	0.600
3.6	Pumps	Pump B Conc.	100
3.7	Pumps	Pump B Conc.	25
4.1	Pumps	Pump B Conc.	25
4.4	Pumps	Pump B Conc.	100
4.9	Pumps	A/B Total Flow	0.600
4.9	Pumps	Pump B Conc.	100
5.0	Pumps	Pump C Flow	0
5.1	Pumps	Pump B Conc.	0
5.1	Pumps	A/B Total Flow	0.400
5.1	Pumps	Pump C Flow	0.100
7.0	Controller	Stop	

## Data Availability

Not applicable.

## Sample Availability

Not available.
